# New Approaches to Microbiome-Based Therapies

**DOI:** 10.1128/mSystems.00122-19

**Published:** 2019-06-04

**Authors:** Andrea C. Wong, Maayan Levy

**Affiliations:** a Department of Microbiology, University of Pennsylvania, Philadelphia, Pennsylvania, USA

**Keywords:** gnotobiotics, metabolites, metagenomics, microbiome, microbiome-based therapies, postbiotics

## Abstract

Over the last decade, our understanding of the composition and functions of the gut microbiota has greatly increased. To a large extent, this has been due to the development of high-throughput genomic analyses of microbial communities, which have identified the critical contributions of the microbiome to human health. Consequently, the intestinal microbiota has emerged as an attractive therapeutic target. The large majority of microbiota-targeted therapies aim at engineering the intestinal ecosystem by means of probiotics or prebiotics. Recently, a novel therapeutic approach has emerged which focuses on molecules that are secreted, modulated, or degraded by the microbiome and act directly on the host. Here, we discuss the advantages and challenges associated with the metabolite-based “postbiotic” approach, highlighting recent progress and the areas that need intensive attention and investigation over the next 5 years. The time is ripe for postbiotic therapies to be developed in the near future.

## Abstract (precis)

Over the last decade, our understanding of the composition and functions of the gut microbiota has greatly increased. To a large extent, this has been due to the development of high-throughput genomic analyses of microbial communities, which have identified the critical contributions of the microbiome to human health.

## PERSPECTIVE

Throughout mucosal surfaces and cavities, the human organism is colonized by a vast community of microorganisms (1). The intestinal microbiota contributes to multiple physiological processes of the host, including metabolic and nutritional homeostasis, immunity, and neuronal activity (2). In turn, the host provides a stable colonization niche for commensal microorganisms and ensures continuous influx of dietary nutrients. With technological advances in genome sequencing (metagenomics) and gnotobiotics (including the use of germ-free mice), the significant contribution of the microbiome to human health has become more apparent. Using these tools, we have expanded our understanding of the interactions between the host and the microbiome over the last decade. Aberrations in the composition and function of the intestinal microbiome have been linked to the molecular etiology of multiple diseases. A number of factors can result in loss of microbial diversity and homeostatic function, including infection, inflammation, diet, xenobiotics, hygiene, and altered host genetics (3). Consequently, efforts are ongoing to devise interventions that aim at modulating the microbiome to improve health.

## MICROBIOME-BASED THERAPIES

Given its extensive role in different gastrointestinal and nongastrointestinal diseases, the microbiome has become an increasingly attractive target for potential therapeutics (4). Nonetheless, one of the biggest challenges in microbiome research is to determine cause-effect relationships and to design microbiome-based therapies that are able to achieve predictable outcomes on the microbial community and host health. The majority of current microbiome-based therapeutics target the prokaryotic arm of the microbiome by aiming to alter the microbial composition of the gut through exogenous administration of live microbes. These approaches, collectively termed probiotics, have become increasingly popular in the last decade. However, there is little evidence to support the efficacy of probiotics. One alternative approach to probiotics is prebiotics. Rather than administering live bacteria, prebiotics are compounds that are consumed with the intention of affecting microbiome composition or function in a beneficial way. Prebiotics, like probiotics, are a relatively unspecific approach to microbiome-based interventions, and further study is needed to fully characterize the effects of prebiotics on different bacterial species.

Currently, a successful FDA-approved microbiome-based intervention is fecal microbiota transplantation (FMT). FMT involves the transfer of an entire microbial community from a healthy donor to a diseased recipient in order to replace the disease-associated microbiome. FMT has been shown to have remarkable efficacy in treating Clostridium difficile infection, which most commonly presents after antibiotic treatment (5). The use of FMT for additional disease conditions is currently being evaluated. Nevertheless, FMTs are associated with substantial risks for the recipient, including the inadvertent transplantation of pathobionts and negative interactions with the recipient’s existing microbial community. Due to the substantial microbial community variability between individuals and the limited long-term stabilization of a foreign microbial configuration, opportunities are plentiful for alternative approaches that are based on a new mechanistic understanding of the microbiome’s involvement in human health.

## METABOLITE-BASED THERAPEUTICS AS ATTRACTIVE ALTERNATIVE TREATMENTS FOR DYSBIOSIS

**FIG 1:**
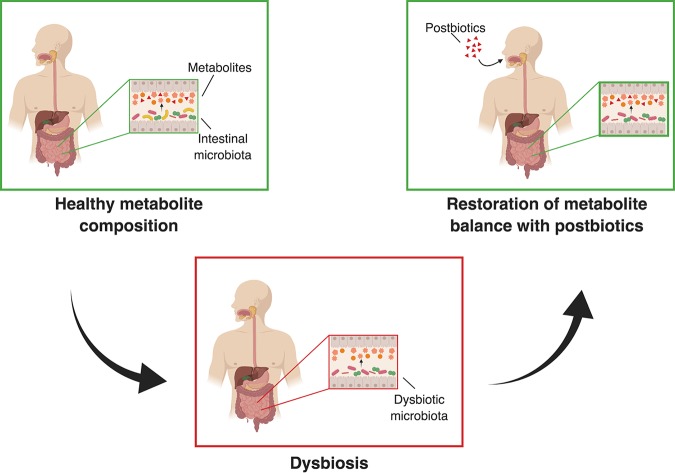
The postbiotic concept. Under homeostatic conditions, the intestinal microbiota produce, modify, and degrade small molecules, collectively termed metabolites, which serve as an effective means of communication in host-microbe interactions and profoundly affect human health. Dysbiosis leads to subsequent alterations in metabolite composition, which has been shown to have direct consequences on host health in the context of multiple diseases. Metabolite-based therapeutics, “postbiotics,” target downstream signaling pathways of the microbiome and act by mitigating the negative effects of an excess, scarcity, or dysregulation of metabolites involved in these pathways. As such, exogenous administration of metabolites has the potential to be an effective therapy against the consequences of dysbiosis.

Research over the past few years has revealed that the intestinal microbial community exerts much of its impact on host physiology through the secretion of small molecules that modulate cellular and organismal functions of the host (6–9). These small molecules serve as an effective means of communication in host-microbe interactions. Metabolite-based therapeutics, or “postbiotics,” target downstream signaling pathways of the microbiome and act by mitigating the negative effects of an excess, scarcity, or dysregulation of metabolites involved in these pathways. As such, rather than targeting the aberrant microbial composition, exogenous administration or inhibition of metabolites has the potential to counteract and correct the negative effects of dysbiosis (Fig. 1).

Initial examples of potential metabolite-based therapies that have been discovered in animal models of human disease include short-chain fatty acids (SCFAs), which were shown to have an anti-inflammatory effect and are altered in inflammatory bowel disease (IBD) patients (10–12); flavonoids that have been implicated in therapies for metabolic diseases (13); and the organic acid taurine, which ameliorates intestinal inflammation (14).

Metabolite-based interventions are therapeutically attractive for several reasons. These small molecules are physiologically abundant at high concentrations, and thus the potential for toxicity is low. In contrast to the administration of live organisms, their dosage and routes of administration follow the principles of pharmacokinetics. Moreover, metabolites are present at most body sides and thus suitable for different routes of administration. Additionally, metabolites are generally stable in the systemic circulation and thus amenable for scalable modulation of their concentration.

Among the downsides of metabolite-based therapeutics are the shorter half-lives than administration of live bacteria; hence, repeated dosing may be required for the treatment of dysbiosis-associated conditions. In addition, the effects of some microbiome-associated metabolites are pleiotropic and highly cell type specific. As such, further characterization of the full effects of different metabolites is necessary in order to understand potential side effects of postbiotics.

## DEVELOPING AREAS OF METABOLITE RESEARCH

The functional effects of microbiome-associated metabolites can broadly be subdivided into two categories: integration into host intracellular metabolism and receptor-mediated metabolite sensing (signaling molecules). In the case of the former, the microbiome functions as an endocrine energy source for other tissues in the body (15). In the latter case, microbiome-derived metabolites can be directly recognized by the eukaryotic cells of the host. Metabolites can trigger receptor-mediated signaling cascades and thereby mediate cell-specific transcriptional responses, which can take place locally in the gastrointestinal tract or even systemically. Such signaling functions have been identified for multiple metabolites and their interaction with the immune system, including SCFAs, niacin, and indole derivatives, among others (9).

We have recently highlighted the concept that both metabolic and signaling functions can be integrated by the same molecules. Metabolites derived from the gastrointestinal community of microorganisms do not only integrate into cellular metabolism but are at the same time essential regulators of intestinal epithelial cell and immune cell function (3). In order to better understand how metabolites affect the host, a systematic effort is required to identify microbiome-derived molecules and the mechanisms by which the host senses and responds to them. One of the mechanisms by which the host is able to detect microbial elements is through pattern recognition receptors (PRRs), such as nucleotide-binding and oligomerization domain-like receptors (NLRs). Recognition of microbial motifs through PRRs can trigger downstream signaling cascades that can result in either antimicrobial responses or tolerance. Previously, using an unbiased metabolomics approach, we have shown that the activity of NLRP6, an important mediator of intestinal immunity, is modulated by microbiome-associated metabolites, such as the organic acid taurine. Importantly, exogenous administration of taurine activated the NLRP6 inflammasome and ameliorated colitis severity (14). Therefore, metabolic and signaling functions of metabolites may be overlapping. A similarly multifaceted role has been identified for the SCFA butyrate, which has been shown both to integrate into cellular metabolism and to serve as a signaling molecule (16).

This complexity raises a further question: What degree of redundancy and complementarity exists between metabolites of a specific biochemical pathway (such as the SCFAs acetate, butyrate, and propionate or the polyamines ornithine, putrescine, and spermine)? Unique and overlapping functions have been described in both cases, and it is currently unknown to what extent metabolites acting in the same pathway may complement each other’s functions or act in a redundant manner (17–19). Additionally, it is conceivable that the host senses multiple products of a single pathway in order to amplify the appropriate response.

A major challenge associated with the postbiotic approach is the pleiotropic function of metabolites. For example, SCFAs have been shown to have a number of effects on human physiology, including gut integrity, metabolic control, appetite regulation, and immune function, which were shown to be associated with improving health (20). Studies of dosage and route of administration will be critical to determine how a specific biological function can selectively be promoted.

Despite the fact that the microbiome is estimated to account for more than half of all fecal and urinary metabolites, only about a dozen metabolites currently have a well-characterized effect on the host, including target cells, receptors, signaling pathways, and physiological outcomes (21). Systematic efforts are required to broaden this spectrum of well-characterized metabolites, using high-throughput technology and defined readout systems. Ultimately, only a comprehensive approach will allow us to address the chemical design principles of metabolite-host interactions. Can metabolite classification by chemical structure tell us something about metabolites’ bioactive function? Do microbiome-derived metabolites cluster into a finite number of groups with similar functional relevance for the host? Will we ultimately be able to predict the biological function of a metabolite based on biochemical relatedness to other metabolites? Answering these questions can yield fundamental insights into the drivers of host-microbial coevolution.

## COEVOLUTION OF INTESTINAL MICROBES AND THE HOST

A closely related aspect of intestinal metabolite biology that can inform therapeutic approaches is the teleology of the most common interactions between host and microbial metabolites. This is most intuitive in the case of essential microbial metabolites, which are compounds that are uniquely produced by the microbiome and cannot be synthesized by the host. For example, while humans cannot degrade complex carbohydrates, intestinal bacteria metabolize these compounds into molecules like SCFAs, which the host can then detect and utilize through a number of different receptors, metabolic pathways, and immune responses (17). As such, metabolites may serve as clear indicators of the activity of the intestinal microbial community. A rise, drop, or relocalization of certain metabolites can serve as a signal to the host that the metabolic activity of the microbial community is altered, potentially an early indication of dysbiosis or bacterial infection. In addition, metabolites serve as nutrients and signaling molecules by which bacteria communicate not only with the host but also with each other. A change in metabolite production may thus be a result of a changing nutrition source, and this change in metabolite signature may allow nearby colonies to anticipate and react to the change. The investigation of evolutionary function of intestinal metabolites is still a wide-open field.

## CONCLUSIONS AND FUTURE DIRECTIONS

Despite its current limitations, a metabolite-based therapeutic strategy is highly promising. In the next 5 years, microbiome researchers will need to include metabolomic characterization of the microbial ecosystem in their routine repertoire of tools, which will allow the community to define functional signatures for disease states that have so far been associated only with compositional and metagenomic changes. Simultaneously, identification of new sensors for microbial metabolites will enable new insights into the mechanisms by which the activity of the microbial ecosystem is assessed by the host, with direct implications for the initiation of inflammation versus the maintenance of homeostasis. The discovery of novel sensors of metabolites will ultimately enable the targeted manipulation of the downstream signaling cascades in cases where the host has a disproportionate response to the microbiome. Metabolite-based therapeutics offer a direct and actionable approach to tackling the effects of dysbiosis on the host. The era of metabolite research in microbiome science has already begun, and concerted efforts may achieve the development of treatments for dysbiosis-associated illnesses for clinical application.
